# Cost-effectiveness of hydroxychloroquine retinopathy screening: the current guideline versus no screening and reduced regimens

**DOI:** 10.1007/s10198-024-01715-w

**Published:** 2024-08-20

**Authors:** Sara W. Quist, Sophie te Dorsthorst, Roel D. Freriks, Maarten J. Postma, Carel B. Hoyng, Freekje van Asten

**Affiliations:** 1 Department of Health Sciences, University of Groningen, University Medical Center, Groningen, The Netherlands; 2 Asc Academics B.V., Groningen, The Netherlands; 3 Department of Ophthalmology, Radboud University Medical Center, Nijmegen, The Netherlands; 4 Department of Health Technology and Services Research, TechMed Centre, University of Twente, Enschede, The Netherlands; 5 Department of Economics, Econometrics & Finance, Faculty of Economics & Business, University of Groningen, Groningen, The Netherlands; 6 University Eye Clinic, Maastricht University Medical Center+, Maastricht, The Netherlands

**Keywords:** Cost-effectiveness, Hydroxychloroquine retinopathy, Screening guidelines

## Abstract

**Objective:**

Hydroxychloroquine (HCQ) effectively treats autoimmune diseases but prolonged use may lead to retinopathy and subsequent vision loss. Guidelines suggest annual follow-up after 5 years for low-risk and 1 year for high-risk patients. This study evaluates the cost-effectiveness of current screening guidelines and a reduced regimen in the Netherlands from a societal perspective.

**Methods:**

A Markov model assessed costs and quality-adjusted life-years (QALYs) for current and reduced screening regimens. The model included 359 HCQ-treated patients from Radboud University Medical Center. Cost-effectiveness was examined in the general population and patients using < 5.0 mg/kg, 5.0–6.0 mg/kg, or > 6.0 mg/kg HCQ per day for several reduced regimens.

**Results:**

Compared to no screening, the current screening guideline saves costs (i.e., €210 per patient), while gaining QALYs (i.e., 0.79 QALY per patient) over a lifetime in the Netherlands. However, in patients receiving < 5.0 mg/kg HCQ per day, a biennial screening regimen after 10 years using SD-OCT was more cost-effective. For those with 5.0–6.0 mg/kg and > 6.0 mg/kg per day, initiating annual screening with an SD-OCT after 5 years was more cost-effective than the current guideline.

**Conclusions:**

Screening for HCQ retinopathy is cost-effective, but delayed initiation and a reduced frequency, using solely an SD-OCT, are more cost-effective. We recommend screening with an SD-OCT and a biennial regimen after 10 years for low-risk patients, an annual regimen after 5 years for intermediate- and high-risk patients.

**Supplementary Information:**

The online version contains supplementary material available at 10.1007/s10198-024-01715-w.

## Introduction

Hydroxychloroquine (HCQ) is a widely used immunomodulatory drug for the treatment of rheumatic disorders such as rheumatoid arthritis (RA) and systemic lupus erythematosus (SLE). While effective, long-term use of HCQ can potentially induce HCQ retinopathy as side effect. HCQ retinopathy is characterized by parafoveal photoreceptor inner or outer segment junction loss and retinal pigment epithelial interruption on spectral-domain optical coherence tomography (SD-OCT) [1]. This can also be recognized as a paracentral scotoma on the visual field by a Humphrey field analyzer (HFA) 10–2 [2]. In persons of Asian descent, retinal toxicity tends to present near the vascular arcades which can be more appropriately visualized by HFA 24–2 [3]. In advanced stages, there is an additional loss of foveal photoreceptors leading to a decline in visual acuity [4]. Autofluorescence imaging can aid the diagnosis, showing a hyperfluorescent ring around the fovea in a bull’s eye pattern [5]. In suspected cases, multifocal electroretinography (mERG) can show paracentral loss of retinal function and confirm the diagnosis of HCQ retinopathy [6].

Retinal damage due to HCQ is often bilateral and irreversible; however, significant loss of function can be prevented by timely discontinuation of HCQ [4]. Guidelines have been developed for the early detection of retinopathy. The Dutch guideline for ophthalmic screening for HCQ retinopathy is based on and equivalent to the American Academy of Ophthalmology (AAO) guideline [7, 8]. The guidelines recommend baseline ophthalmic screening, followed by annual screening after 5 years of use in low-risk patients and after 1 year in high-risk patients [8]. High risk is considered an HCQ dose of more than 5.0 mg/kg/day, simultaneous use of tamoxifen, and concomitant renal insufficiency. Screening is performed using spectral-domain optical coherence tomography SD-OCT and HFA [8].

The prevalence of RA is approximately 0.5% [9] and it is estimated that about 16% of people with RA use HCQ [10]. Effectively, this adds up to approximately 14,000 individuals on HCQ in the Netherlands alone. Consequently, annual screening constitutes a large burden on ophthalmology departments, while already experiencing high pressure. To illustrate, the waiting lists in ophthalmology are the third longest of all specialties in the Netherlands with 12.5 weeks on average in 2023 (i.e., 8.5 weeks longer than the agreed maximum standard) [11]. Moreover, the annual Dutch healthcare costs of eye-related illnesses are significant and sum up to approximately 1 billion euro [12]. To reduce the strain on ophthalmic departments, efficiency should be improved, and unnecessary clinic visits reduced.

Although current guidelines are effective, a comprehensive evaluation of its cost-effectiveness has not been conducted to date. Cost-effectiveness analyses are used to weigh costs against effects, usually defined in terms of quality-adjusted life-years (QALYs), in order to optimize efficiency in healthcare strategies. The issue of increasing (ophthalmic) healthcare burden and the search for optimization is relevant for healthcare systems in many countries. Therefore, in this study, we evaluate the cost-effectiveness of the current guideline for screening for HCQ retinopathy from a Dutch societal perspective and assess whether cost-effectiveness can be improved.

## Methods

### Study characteristics

Our study determined the cost-effectiveness of the current screening guideline in the Netherlands for patients that are chronically treated with HCQ from a Dutch societal perspective, according to the Consolidated Health Economic Evaluation Reporting Standard 2022 (Cheers 2022) (Supporting file 1) [13]. The model calculated the healthcare-related and societal costs and QALYs based on the number of retinopathy cases of the current screening guideline compared to no screening and several reduced screening regimens. The incremental costs and QALYs were used to calculate the incremental cost-effectiveness ratio (ICER) of the current screening guideline over a lifetime horizon (i.e., 54 years) compared to no screening. We applied a willingness-to-pay threshold of €20,000, therefore, in case the ICER fell below €20,000, the screening regimen was considered cost-effective [14]. First, the study assessed the cost-effectiveness of current screening guideline compared to no screening. This comparison was relevant because the cost-effectiveness of screening has not yet been firmly established. Second, the study explored whether the screening burden could be reduced without compromising the effect of screening. This was done by comparing multiple reduced screening regimens tailored to individual patient risk with the current screening guideline.

### Current screening guideline

Currently, patients that start chronic HCQ treatment are recommended to receive SD-OCT and HFA screening in the first year of treatment initiation, followed by a yearly screening after 5 years of use in low-risk patients and after 1 year in high-risk patients [7, 8]. High-risk patients are defined as patients with renal insufficiency (i.e., estimated glomerular filtration rate [eGFR] < 60 ml/min), patients that use tamoxifen, and patients that receive an HCQ dosage above 5.0 mg/kg per day. Although evidence is inconclusive, in general patients that have some form of maculopathy or retinopathy at baseline are regarded as high-risk [8].

### Reduced screening regimens

In addition to evaluating the cost-effectiveness of the current screening guideline for the general population compared to no screening, our study aimed to identify more cost-effective screening regimens for different risk groups based on the daily dosage of HCQ. The risk groups were defined as follows: patients receiving < 5.0 mg/kg, 5.0–6.0 mg/kg, and > 6.0 mg/kg. Due to limited data, we did not perform a subgroup analysis for patients with renal insufficiency or using tamoxifen.

**Fig. 1:**
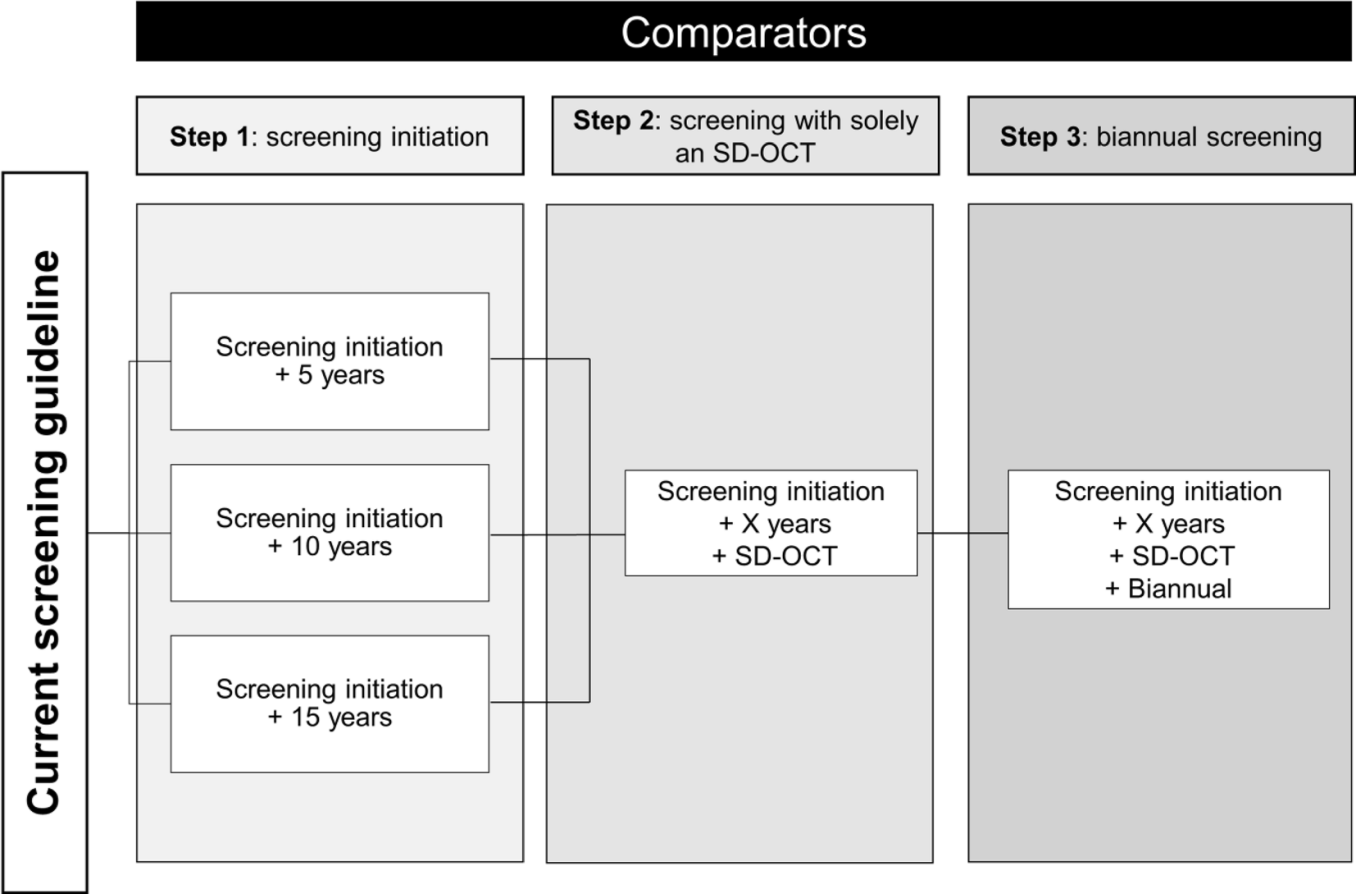
Overview of different reduced screening regimens. Key: First, the impact of a later time of screening initiation is assessed. Second, the use of solely an SD-OCT in combination with different times for screening initiation is explored. Third, the combination of later screening initiation, solely an SD-OCT, and biennial (i.e., every two years) screening is explored. The reduced screening regimens are assessed in 3 risk groups: patients receiving < 5.0 mg/kg, patients receiving 5.0–6.0mg/kg, and patients receiving > 6.0mg/kg HCQ per day. *SD-OCT* spectral-domain optical coherence tomography

Several reduced screening regimens were proposed (Fig. 1). First, we examined the effect of delaying the initiation of annual screening by using five-year intervals. In this regimen, patients were still screened in the first year after treatment initiation but the regular preventive screening was delayed by five-year intervals. Second, we explored the impact of different initiation times for screening combined with the use of SD-OCT as the sole screening method. Finally, we assessed the impact of a delayed initiation time, exclusive use of SD-OCT for screening, and replacing annual with biennial screening.

We used a step-wise comparison method for each risk group to identify the most cost-effective screening regimen, that limited the required resources while maximizing induced QALYs. We identified the total costs and QALYs for the proposed regimens and sorted them from lowest to highest cost. The least expensive regimen was compared to the current screening guideline. Then, the least expensive proposed regimen was compared to the second least expensive option. If the was ICER below the WTP-threshold of €20,000 per QALY, the next least expensive option was compared to the current most cost-effective option. This process was repeated until an ICER above €20,000 was found, indicating that the previous regimen was most cost-effective.

### Model structure

**Fig. 2:**
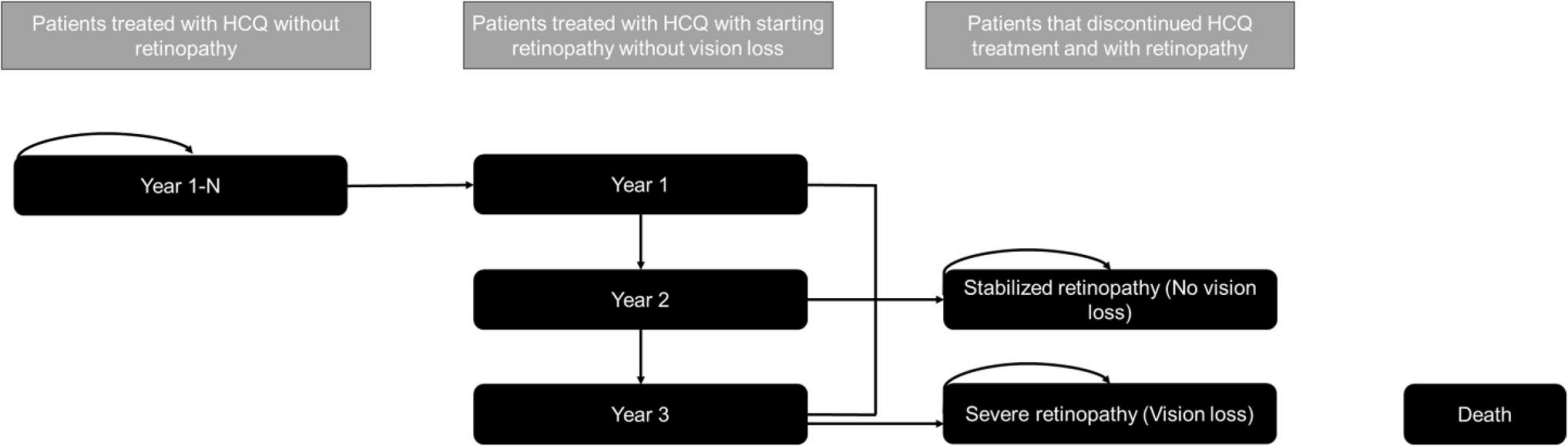
Model structure. Key: All patients started with HCQ treatment without retinopathy but were at risk to develop retinopathy. Following each year during which a patient remained free of retinopathy, they transitioned to a new health state with an escalated risk for retinopathy. In case a patient developed retinopathy, the patient progressed to a ‘starting retinopathy without vision loss’ health state. If the patient’s retinopathy was not detected within 3 years, the patient developed severe retinopathy with vision loss. If the retinopathy was detected within 3 years, the patient was assumed to have stabilized retinopathy without vision loss. After HCQ discontinuation, the retinopathy could not further deteriorate. *HCQ* Hydroxychloroquine

A Markov cost-effectiveness model was developed in Microsoft Excel 2016 (Redmond, WA, USA) to mimic the screening pattern and disease pathway of patients in their best-seeing eye (Fig. 2). The model used a 1-year cycle length, in line with the screening frequency and endorsed by the slow progression of retinopathy [15]. All patients started with the first year of HCQ screening with a risk for developing retinopathy (i.e., the health state ‘Treated with HCQ’). The risk for retinopathy increased over the years and was, therefore, dependent on the year of treatment. In case a patient developed retinopathy, the patient transitioned to a ‘starting retinopathy without vision loss’ health state. If the retinopathy was not detected within 3 years, the patient was assumed to develop severe retinopathy, characterized by severe vision loss in both eyes. If retinopathy was detected within the first 2 years, the patient was assumed to have stabilized retinopathy without vision loss after discontinuation of HCQ treatment. There was no possibility of deterioration of retinopathy after discontinuation of HCQ treatment. The 3-year assumption was based on literature and expert opinion. Due to the discontinuation of HCQ after early retinopathy detection, it is challenging to estimate how long it takes for the structural damage to progress into vision loss. A recent study showed how prior to the onset of typical SD-OCT structural changes, there is a period of 3–6 years of macular thinning [16]. This implies a gradual deterioration over the course of several years and the assumption of vision loss after 3 years of retinopathy going undetected seems a conservative estimate. In the literature, we identified only one case of HCQ retinopathy where the patient did not stop HCQ [17]. After detection of early retinopathy changes, the patient was lost to follow-up for 7 years, after which they developed advanced retinopathy. It was not reported whether this resulted in loss of visual acuity [17]. Death was an absorbing health state and was based on the background mortality of the Dutch population as it was assumed that severe retinopathy did not affect mortality.

### Patient characteristics

The model explored screening regimens for the general population treated with HCQ, patients receiving < 5.0 mg/kg HCQ per day, patients receiving 5.0–6.0 mg/kg HCQ per day, and patients receiving > 6.0 mg/kg per day. Patient characteristics were based on data from the Radboud university medical center (Nijmegen, Netherlands) to reflect the Dutch general population. This research was submitted to the local committee on research involving human subjects and this committee ruled that approval was not required for this study. We assembled data from all patients that were known to use HCQ and that had visited the Department of Ophthalmology of the Radboud university medical center for screening at least once between 2014 and 2019 (n = 354) (Table 1). As the identified incidence for HCQ retinopathy was low (i.e., 4 in 354 patients) and was not in line with previous literature based on larger patient samples [18], the risk for retinopathy was based on existing data instead of the internal data of the Radboud university medical center. The percentage of male patient and average weight of the patients from Radboud University Medical center was comparable with the patient characteristics of the patients in the study of Melles et al. [18]. In addition, in the population in the study of Melles of et al. 12.0% of patients had renal insufficiency and 0.4% used tamoxifen, whereas in the Radboud population 13% had renal insufficiency and 0% of patients used tamoxifen. The average age in the Radboud population was slightly lower than in the study of Melles et al. (i.e., 46.2 years versus 58.2 years).

**Table 1:** Patient characteristics

	Input
Total number of screened patients	354
Average age at start HCQ, years (SD)	46.2 (18.6)
Male, n (%)	83 (23%)
Visual acuity best-seeing eye at start HCQ, LogMAR (SD) (n = 56)	0.07 (0.11)
Tamoxifen use, n (%)	0 (0%)
Kidney dysfunction (eGFR below 60 ml/min), n (%)	46 (13%)
Average weight (SD)	73.8 (18.2)
Dosage, n (%)	
< 5.0 mg/kg per day	203 (57%)
5.0–6.0 mg/kg per day	66 (19%)
> 6.0 mg/kg per day	85 (24%)
Indication, n (%)	
Systemic lupus erythematosus	129 (36%)
Rheumatoid arthritis	104 (29%)
Other indication	121 (34%)
Years of follow-up, n (%)	
0–2 years	57 (16%)
2–5 years	60 (17%)
5–10 years	134 (38%)
> 10 years	103 (29%)

*eGFR* estimated glomerular filtration rate, *LogMAR* logarithmic minimum angle of resolution, SD: Standard deviation

### Transition probabilities

The transition between health states was based on the cumulative risk for HCQ retinopathy that was found by Melles et al. and the sensitivity of screening (Table 2) [15, 19]. The risk for HCQ retinopathy depended on the dosage of patients (i.e., < 5.0 mg/kg, 5.0–6.0 mg/kg, > 6.0 mg/kg per day) and the year of treatment (Supporting file 2) [19]. The study by Melles et al. estimated the risk of retinopathy over 15 years, and to account for risk over a lifetime horizon, polynomial regression was performed in RStudio (Supporting file 2). Using the cumulative risk, the yearly risk was calculated based on the year of treatment. The regression was performed on the extracted data from the Kaplan–Meier curve, using WebPlotDigitizer. Polynomial regression was found to be the most fitting out of several linear, non-linear, Bayesian, and survival models. The sensitivity of screening was dependent on the instrument(s) used and was based on the literature [15]. As retinopathy does not impact the mortality of patients, solely background mortality was considered in the model, based on data from Statistics Netherlands [20].

**Table 2:** Input parameters used to calculate the transition probabilities

Parameter	Input	Source
Risk for HCQ retinopathy	Dependent on years of use	Supporting file 2, Melles et al. [19]
Sensitivity of screening with HFA and SD-OCT	85.7%	Yusuf et al. [15]
Sensitivity of screening with SD-OCT	78.6%	Yusuf et al. [15]

*HCQ* Hydroxychloroquine, *HFA* Humphrey field analyzer, *SD-OCT* spectral-domain optical coherence tomography

Utility values.

We based our effects on the number of prevented retinopathy cases and calculated the impact on patients' quality of life of (severe) retinopathy. QALYs are life years corrected for their quality-of-life based on patients’ preference, expressed in utility scores. To determine patients’ utility scores, we considered their visual acuity in best-seeing eye and age and used time trade-off values from a regression analysis described in literature (Supporting file 3) [21]. We assumed that retinopathy affects patients’ quality of life in a similar way to macular degeneration due to similarities in affected retinal location [15, 22]. The LogMAR of the best-seeing eye at model initiation was 0.07 (Snellen 20/23) according to patient data from the Radboud university medical center (Table 1). The study of Allahdina et al. demonstrated that early to moderate retinopathy did not impact visual acuity [23], and therefore, we assumed the same LogMAR for patients with stabilized retinopathy. As retinopathy affects both eyes symmetrically, the LogMAR in the best-seeing eye of patients with severe retinopathy was set at 0.7 (Snellen 20/100) [18, 23]. Because a LogMAR of 0.7 already represents the most severe form of retinopathy, we assumed this LogMAR value remained stable after diagnosis. In an additional analysis, the impact of the visual acuity associated with severe retinopathy was assessed. All QALYs were discounted at 1.5% per year [14].

### Costs

The model included both costs within the healthcare system (i.e., medical costs) and costs for patients and caregivers (i.e., societal costs) (Table 3) [14]. All costs were expressed in €, inflated to March 2023 prices based on the consumer price index of Statistics Netherlands [24], and discounted with a rate of 4.0% per year [14].

**Table 3:** Costs inputs and their implementation frequency for costs within the healthcare system and for patient and care giver

Parameter	Value	Implementation frequency	Source
Costs within the healthcare system			
OCT + HFA	€206	Screening regimen for preventive screening Yearly screening after development of retinopathy	DBC: 079699011, internal data
OCT	€154	Screening regimen for preventive screening Yearly screening after development of retinopathy	DBC: 079699011 NZA[25, 30]
Vision aids associated with a visual acuity of LogMAR 0.7 (Snellen 20/100)	€1,301	1 time after development of severe retinopathy	Internal data
Costs for patient and care giver			
Productivity loss due to screening	For SD-OCT and HFA screening: 1 h For SD-OCT screening: 0.5 h Workforce male (45–75 years): 67% Workforce female (45–75 years): 56% Hourly wage: €45	Screening regimen for preventive screening for patients below 67 Yearly screening after the development of severe retinopathy for patients below 67	Internal data, CBS, Dutch cost manual [25]
Productivity loss due to severe HCQ retinopathy	Percentage of patients that stopped working: -5.5% Hourly wage: €45	For 85 days after developing severe retinopathy	Brown et al. [28] Expert opinion Dutch cost manual [25]
Informal care due to severe HCQ retinopathy	Yearly hours: 220 Hourly opportunity costs: €18	Yearly after severe retinopathy	Brown et al. [28] Expert opinion Dutch cost manual [25]
Travel costs	Average distance to the hospital: 7 km Costs per km: €0.24	Every screening	Dutch cost manual

*HCQ* Hydroxychloroquine, *HFA* Humphrey field analyzer, *LogMAR* logarithmic minimum angle of resolution, *SD-OCT* spectral-domain optical coherence tomography

Screening costs were implemented for every preventive screening and screening after retinopathy diagnosis. Patients received annual screening after both stabilized retinopathy without vision loss and after severe retinopathy with vision loss. The costs of screening were based on Dutch literature and the Dutch cost manual [25–27]. If a patient developed severe retinopathy with vision loss, costs for vision aids used by patients with a LogMAR of 0.7 in best-seeing eye were also considered, which was based on internal data of the Radboud university medical center provided by Low Vision Totaal.

Costs for patients and caregivers included productivity losses due to (preventive) screening, productivity losses due to vision loss after severe retinopathy, informal care due to severe retinopathy, and travel costs [25]. Productivity losses were calculated using the friction cost method, as described in the Dutch costing manual [25]. Hours of productivity losses and informal care were based on literature and validated by a medical expert [28]. To account for productivity losses due to severe retinopathy, 5.5% of patients below the age of 67 (i.e., Dutch retirement age) were assumed to stop working after diagnosis [28]. The maximum of days of productivity loss was set at 85 days after diagnosis, following Dutch guidelines [25, 28]. For every screening moment, 0.5–1 h of productivity losses were considered for patients below the age of 67, which was corrected for the average Dutch working force [29]. The informal care needed because of severe vision loss was estimated to be 220 annual hours [28]. Those hours were valued by using the hourly costs for the replacement of home care [25, 28]. Travel costs were incorporated for every screening visit [25].

Sensitivity and scenario analyses.

A deterministic sensitivity analysis (DSA), probablistic sensitvity analysis (PSA), and scenario analysis were performed for the general population compared to no screening [31]. In the DSA, the impact of the input parameters on the ICER was assessed by varying them between the lower bound of 2.5% and the upper bound of 97.5% of their confidence intervals (CI) based on their standard errors, or within clinically relevant ranges. The PSA was performed to determine model robustness. Input parameters were simultaneously varied for 1,000 simulations using their uncertainty ranges based on their standard errors and respective distributions. Depending on the input parameter normal, beta, and gamma distributions were used (Supporting file 5). A standard error of 25% from the deterministic value was used when the standard error or 95% CI was not available. Several assumptions were made (Supporting File 4). To assess their impact on the model outcomes a scenario analysis was performed. The scenario analysis assessed the use of a healthcare payer’s perspective and 0–10% discounting of costs and effects. In addition, due to the uncertainty around the LogMAR visual acuity after the development of severe retinopathy, an additional analysis was performed that established the impact of this LogMAR value on the incremental QALYs of different screening regimens.

## Results

### Cost-effectiveness of screening guideline in the general population

Our analysis demonstrates that the current screening regimen results in the prevention of 230 cases of severe retinopathy per screening of 1000 patients in the general population over a lifetime horizon (Table 4). Consequently, the current screening guideline results in 22.44 QALYs per patient over a life-time horizon, while no screening results in 21.65 QALYs per patient. The total costs for the current screening guideline amount to €4,112 per patient over a lifetime horizon, while the costs for no screening are €4,322. Therefore, the Dutch screening guideline leads to a gain of QALYs (i.e., 0.79 QALY per patient) and saves costs (i.e., -€210 per patient), resulting in a dominant ICER (Table 4). Preventive screening accounts for most costs in the current screening program (i.e., €3,614 per patient). In the case of no screening, severe retinopathy is the only contributor to total costs, with most costs attributed to the patients and caregivers (i.e., €3,808 out of the total of €4,322).

**Table 4:** Costs per patient per category, incremental costs, incremental QALYs, and effects in the form of cases of severe retinopathy/1000 patients of the current screening regimen and no screening for the general population

General population (outcomes per patient)			
	Current screening	No screening	Difference
Total effect (cases of severe retinopathy/1000 patients)	3	233	230
Total effect (QALYs/patient)	22.44	21.65	0.79
Costs within the healthcare system			
Preventive screening	€3,252	€0	€3,252
Retinopathy without vision loss	€385	€0	€385
Severe retinopathy	€11	€514	-€503
Costs for patient and caregiver			
Preventive screening	€362	€0	€362
Retinopathy without vision loss	€16	€0	€16
Severe retinopathy	€84	€3,808	-€3,723
Total costs	€4,112	€4,322	-€210
ICER	Current screening guideline is dominant over no screening		

*ICER* Incremental cost-effectiveness ratio, *QALYs* Quality-adjusted life-years

### Reduced screening regimens per risk group

Additionally, we evaluated the cost-effectiveness of the current screening guidelines per risk group and identified the most cost-effective screening regimen using a stepwise comparison (supporting file 6).

The stepwise comparison showed that for patients receiving < 5.0 mg/kg/day, a biennial screening regimen after 10 years with solely an SD-OCT is most cost-effective (Table 5). It reduces the costs of the current screening guideline from €3,710 to €1,751 (i.e., -€1,959), while lowering QALYs from 22.46 to 22.34 (i.e., -0.12). In patients with receiving 5–6 mg/kg/day, a reduced screening regimen with a screening initiation after 5 years and use of solely an SD-OCT is most cost-effective. This proposed regimen reduces costs from €3,222 to €2,953 (i.e., -€269), while reducing QALYs from 22.43 to 22.40 (i.e., -0.03). In patients receiving > 6 mg/kg/day, screening initiation after 5 years and use of solely an SD-OCT is cost-effective. It reduces costs from €4,686 to €3,194 (i.e., -€1,492) and QALYs from 22.41 to 22.36 (i.e., -0.05).

**Table 5:** Overview of the total costs and QALYs of the current screening guideline and most cost-effective screening regimen for patients that receive < 5.0 mg/kg HCQ per day, 5.0–6.0 mg/kg HCQ per day, and > 6.0 mg/kg HCQ per day

	Current screening guideline		Proposed screening regimen		
	Total costs	Total QALYs	Proposed option	Total costs	Total QALYs
Patients receiving < 5.0 mg/kg HCQ per day	€3,710	22.46	10 years increment, biennial, SD-OCT only	€1,751	22.34
Patients receiving 5.0–6.0 mg/kg HCQ per day	€3,222	22.43	5 years increment, SD-OCT only	€2,953	22.40
Patients receiving > 6.0 mg/kg HCQ per day	€4,686	22.41	5 years increment SD-OCT only	€3,194	22.36

### Probabilistic sensitivity analysis

**Fig. 3:**
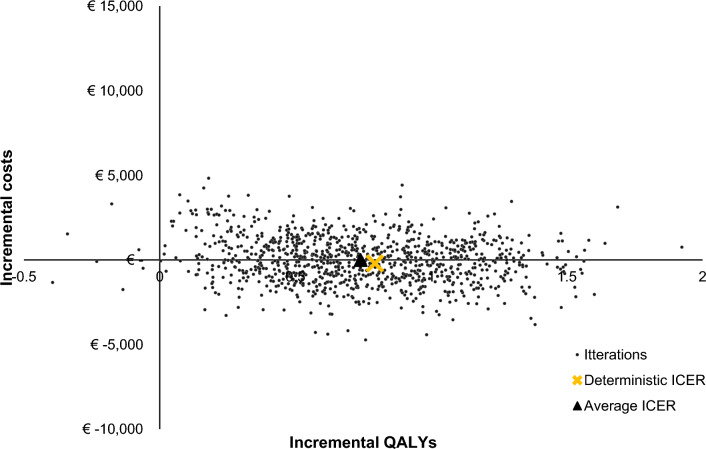
Cost-effectiveness plane of screening compared to no screening in the general population. Key: The red dot represents the ICER that was found in the base case (i.e., incremental costs: -€210 and incremental QALYs: 0.79). The yellow dot represents the average ICER that was found in the PSA (i.e., incremental costs: €20 and incremental QALYs: 0.75). *HCQ* Hydroxychloroquine, *ICER* incremental cost-effectiveness ratio

The cost-effectiveness plane presents the outcomes of the PSA for the current screening guideline compared to no screening in the general population (Fig. 3). The PSA determines that the average incremental QALYs value is 0.74 and the average incremental costs value is -€8. Incremental QALYs range from -0.39–2.14 and incremental costs range from -€4,819 and €4,726. The analysis shows that screening had a 98.7% to be cost-effective with a WTP-threshold of €20,000.

### Deterministic sensitivity analysis

Because the base case analysis of the current screening guideline in the general population resulted in a negative ICER, the DSA was performed separately for the incremental QALYs and costs for the current screening guideline compared to no screening (Supporting file 7). The average age has the most impact on both incremental costs and incremental QALYs. Age reduction to 27.6 leads to incremental QALYs of 1.64 and incremental costs of -€2,377. Other parameters with substantial impact on the incremental QALYs include the QoL correction for vision loss (0.43–1.19 for a correction ranging from 0.21 to 0.56 per LogMar), LogMAR visual acuity of patients with severe retinopathy (0.54–1.04 for a LogMAR ranging from 0.50 to 0.90), and LogMAR visual acuity of patients without retinopathy (0.66–0.88 for a LogMar ranging from 0 to 0.18). Parameters with substantial impact on the incremental costs include the hourly wage of the caregiver (-€1,355 -€2,205 for a for a hourly wage ranging from €8 to €21), yearly hours of informal care (-€1,355 -€2,205 for yearly hours ranging from 126 to 340), and costs for SD-OCT and HFA screening (i.e., -€1,627 -€1,597 for costs per screening ranging from €100 to €270).

### Scenario analysis

The scenario analysis assessed the impact of the exclusion of costs for patient and family (i.e., healthcare payer’s perspective), a 0% and 10% and discounting rate for costs and effects (Table 6). The current screening guideline proves to be cost-effective in all scenarios but was solely cost saving in the scenario that applied a discount rate of 0%.

**Table 6:** Incremental costs, incremental QALYs, and ICER of the current screening regimen compared to no screening in the general population

	Incremental costs	Incremental QALYs	ICER
Base case	−€210	0.79	Dominant
Scenario 1: Healthcare payer’s perspective	€514	0.79	€3,954
Scenario 3: 0% discounting rate	−€6,054	1.28	Dominant
Scenario 3: 10% discounting rate	€953	0.08	€13,225

*ICER* incremental cost-effectiveness ratio

### Analysis of visual acuity due to severe retinopathy

**Fig. 4:**
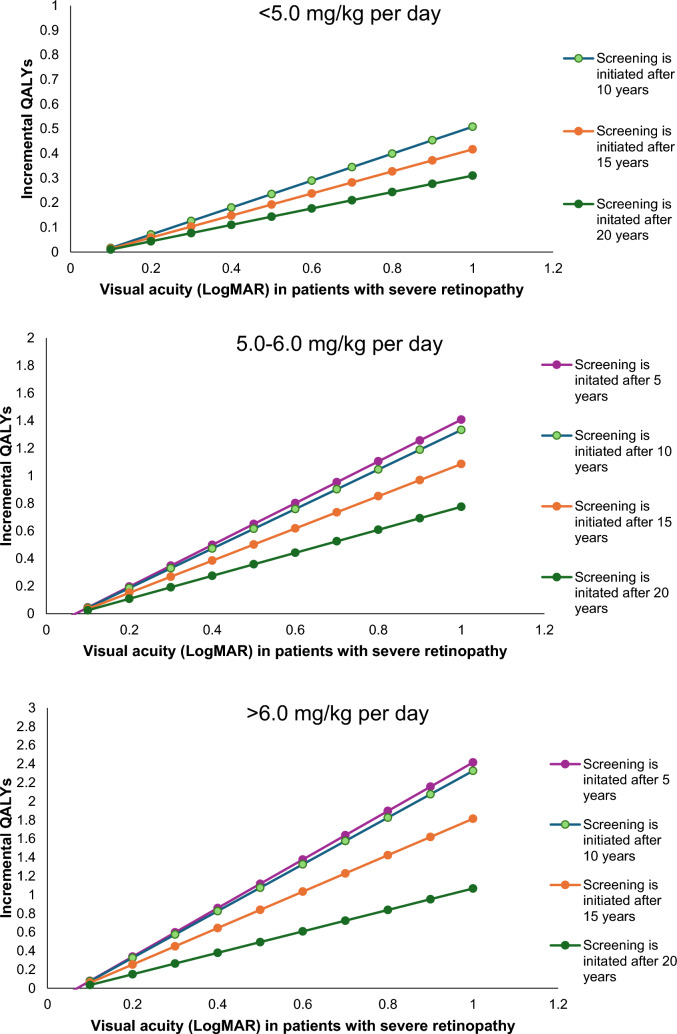
Effect of LogMAR on QALYs gained per screening initiation interval in patients with severe retinopathy, treated with < 5 mg/kg/day, 5–6 mg/kg/day, and > 6 mg/kg/day, compared to no screening. *LogMAR* logarithm of the minimum angle of resolution, *QALY* quality-adjusted life-year

The DSA already showed the considerable impact of LogMAR in best-seeing eye on the incremental QALYs. Additionally, Fig. 4 provides a detailed overview of the impact of visual acuity in patients with severe retinopathy on the found incremental QALYs of reduced screening guidelines versus no screening, categorized by risk groups. The figure illustrates that a less severe LogMAR corresponds to a lower estimated impact of delayed screening initiation compared to no screening. Furthermore, the steeper lines indicate that the effect of visual acuity is larger for the screening regimens initiated after 5 to 10 years compared to those initiated after 15 to 20 years. In a regimen where screening was initiated after 20 years, a LogMAR visual acuity of 0.02 (Snellen 20/21) results in incremental QALYs of 0.01, 0.02, and 0.03, respectively, compared to no screening, in patients receiving < 5.0 mg/kg per day, 5.0–6.0 mg/kg per day, and > 6.0 mg/kg per day. The incremental QALYs increase to 0.3, 0.8, and 1.01 in the same patient groups when a LogMAR visual acuity of 1.0 (Snellen 20/200) is used. In a regimen where screening was initiated after 10 years, a LogMAR visual acuity of 0.2 (Snellen 20/21) induces incremental QALYs of 0.02, 0.04, and 0.08 in patients receiving < 5.0 mg/kg per day, 5.0–6.0 mg/kg per day, and > 6.0 mg/kg per day, respectively. When a LogMAR visual acuity of 1.0 (Snellen 20/200) is used, the incremental QALYs increase to 0.5, 1.3, and 2.3 in the same patient groups.

## Discussion

The strain on ophthalmology clinics is substantial; this is reflected in the long waiting lists and considerable healthcare expenses [11, 12]. To contribute to improved resource allocation in ophthalmology healthcare, our study assessed the cost-effectiveness of the current guideline for screening for HCQ retinopathy and of screening regimens with reduced capacity. We find that the current screening guideline for HCQ retinopathy gains QALYs and saves costs compared to no screening in the general population. However, fewer screening moments and the use of fewer instruments reduces capacity and costs, while having limited impact on effect. Our study showed that in patients receiving < 5.0 mg/kg HCQ per day, extending the time to screening initiation to 10 years instead of 5 years, use of solely an SD-OCT, and biennial screening is more cost-effective than the current screening guideline. For patients receiving 5.0–6.0 mg/kg or > 6.0/mg/kg HCQ per day, deferring the current screening guideline to screening with solely an SD-OCT and initiation after 5 years instead of 1 year is more cost-effective than current screening guideline. To illustrate the impact of a reduced screening regimen in the Radboud University Medical center, an alternative screening regimen in patients receiving < 5.0 mg/kg/day would result in cost-savings of €397,677 (n = 203). For patients receiving 5–6 mg/kg/day and > 6 mg/kg/day, an alternative screening regimen would save the department €17,754 and €126,820, respectively (n = 66 and 85).

Although we approached the objective from a Dutch healthcare perspective, our recommendations can hold value for other countries as well. The Dutch guidelines for HCQ screening are in fact based directly off of the AAO guidelines [7, 8]. The data regarding the risk for HCQ retinopathy were derived from a cohort study conducted in the United States [19] and the clinical characteristics of the hospital population in the Radboud University Medical Center closely corresponded with those of the population in this cohort study. While the United States population contained more patients with RA, we do not expect this to impact model outcomes; factors with a larger impact on outcomes (i.e., age, kidney function, and weight) were similar [19]. Nevertheless, it would still be insightful to conduct a similar analysis using cost and clinical data specific to different countries.

Our study was designed to optimally reflect clinical practice. However, as HCQ retinopathy is a rare disorder and consequently difficult to study, this leads to an inevitable mix of data sources and assumptions. The analysis mostly used the patient information of the Radboud university medical center, however, the retinopathy incidence in this dataset was strikingly low and the risk for retinopathy had to be retrieved from a larger data analysis in literature [19]. No data was available regarding the risk for retinopathy after 15 years of use. To overcome this issue, our analysis extrapolated the risk for retinopathy, leading to some uncertainty in our model. The DSA and PSA confirmed the robustness of our model regardless.

In addition, our study has identified some gaps in the current knowledge of HCQ retinopathy. There is remarkably little data regarding the progression rate of HCQ retinopathy, and therefore, we opted for a conservative model structure that assumed 3 years of undetected retinopathy before experiencing vision loss. Literature suggests that macular thinning typically occurs over 3–6 years, implying that our 3-year period before vision loss is the minimum [16, 17]. Consequently, our model would favor earlier and more frequent screening since the time to severe vision loss is short. Moreover, no recent QoL data of patients with macular degeneration was available and no literature reporting the QoL of patients with HCQ retinopathy was found. Given the similarities in disease pathology and macular location of HCQ retinopathy and age-related macular degeneration, we assumed that the QoL of patients was similar [15, 22]. Another assumption leading to conservative estimates involved the impact of severe retinopathy; Our model structure solely incorporated the most severe form of retinopathy for which we used a stable visual acuity of 0.7 LogMAR. This poor visual acuity was based on a case study reporting the visual acuity of 6 patients with the most advanced form of retinopathy, the largest case series of severe retinopathy so far [23]. As other studies found a substantially better visual acuity for patients with retinopathy (i.e., a LogMAR around 0.3 in patients with severe disease), this assumption might have overestimated the impact of retinopathy on the quality of life [18]. The sensitivity analysis showed that a lower LogMAR led to considerably different estimates of incremental QALYs and would, therefore, lead to a lower difference in incremental QALYs between the current screening guideline and the reduced screening regimens. Likely, our estimates and thus our recommendations are conservative, and we expect the real-world consequence of a reduced screening regimen to be more cost-saving and less impactful on QALY than presented here. As with all models, it is important to reassess the optimal screening regimen once more data regarding retinopathy progression and impact is available.

We did not consider all risk factors that are important for HCQ retinopathy. Literature shows that, apart from dosage, renal failure and tamoxifen use are 2 important risk factors for the development of HCQ retinopathy [18]. Unfortunately, limited data were available about the impact size of those risk factors, and therefore, their inclusion would have led to too much uncertainty in the outcomes. Currently, tamoxifen use and renal failure are considered in the screening guideline [8], and it remains important to consider those risk factors in future guidelines and studies. Therefore, as of this moment we recommend following the current guideline regarding patients on tamoxifen and those with renal failure.

Lastly, while our study focused on the impact of HCQ on retinopathy, it did not consider the beneficial effects of chronic HCQ use in patients with rheumatic disorders. Discontinuation of HCQ after the development of HCQ retinopathy might impact the progression of those disorders. We chose to exclude the impact of HCQ discontinuation, to focus on the ophthalmological practice and to reduce the model complexity. In the end, whether HCQ should be discontinued is a decision the patient should make together with their ophthalmologist and rheumatologist, considering both the aspect of developing retinopathy and the risk of disease activation.

One earlier study was identified that analyzed the cost-effectiveness of screening for HCQ retinopathy in the United States [32]. Although this study uses a different perspective, model type, and solely includes direct medical screening costs, the study endorses our outcomes as it finds screening to be cost-effective. The study also concludes that cost-effectiveness improves with a later screening initiation and in patients with high risk.

A notable strength of our study lies in its comprehensive approach towards evaluating the cost-effectiveness of the present screening guideline, as well as providing practical recommendations for its optimization from a societal perspective. Such an approach is essential in improving healthcare policy-making and resource allocation in ophthalmology [11]. Additionally, through the presentation of outcomes from all evaluated reduced screening regimens, readers can formulate their own conclusions regarding the optimal screening regimen. This model can serve as an example for other countries wishing to optimize their screening regimen.

In conclusion, screening for HCQ retinopathy is cost-effective, however delaying the initiation of screening based on the dosage of a patient and use of solely an SD-OCT will help to reduce costs and capacity, while having minimal impact on the effect. Therefore, we recommend a new screening regimen exclusively utilizing SD-OCT for all patients. For low-risk individuals, screening should commence after 10 years and be conducted biennially. In moderate-risk and high-risk individuals, screening should start after 5 years and be performed annually.

## Supplementary Information

Below is the link to the electronic supplementary material.

Supplementary file1 (DOCX 19 KB)

Supplementary file2 (DOCX 23 KB)

Supplementary file3 (DOCX 13 KB)

Supplementary file4 (DOCX 13 KB)

Supplementary file5 (DOCX 15 KB)

Supplementary file6 (DOCX 25 KB)

Supplementary file7 (DOCX 22 KB)

## Data Availability

Most data are included in the manuscript and its supporting information files. The analyses were largely conducted based on publicly available information which is presented and referenced in the article and supplementary information files. Some of the data generated during and/or analysed during the current study are not publicly available but are available from the corresponding author on request.

## References

[1] Kellner, S., Weinitz, S., Kellner, U.: Spectral domain optical coherence tomography detects early stages of chloroquine retinopathy similar to multifocal electroretinography, fundus autofluorescence and near-infrared autofluorescence. Br. J. Ophthalmol. **93**, 1444–1447 (2009). 10.1136/bjo.2008.157198 19692385 10.1136/bjo.2008.157198

[2] Mavrikakis, M., Papazoglou, S., Sfikakis, P.P., Vaiopoulos, G., Rougas, K.: Retinal toxicity in long term hydroxychloroquine treatment. Ann. Rheum. Dis. **55**, 187–189 (1996). 10.1136/ard.55.3.187 8712882 10.1136/ard.55.3.187 PMC1010126

[3] Melles, R.B., Marmor, M.F.: Pericentral retinopathy and racial differences in hydroxychloroquine toxicity. Ophthalmology **122**, 110–116 (2015). 10.1016/j.ophtha.2014.07.018 25182842 10.1016/j.ophtha.2014.07.018

[4] Marmor, M.F., Hu, J.: Effect of disease stage on progression of hydroxychloroquine retinopathy. JAMA Ophthalmol. **132**, 1105–1112 (2014). 10.1001/jamaophthalmol.2014.1099 24922444 10.1001/jamaophthalmol.2014.1099

[5] Kellner, U., Renner, A.B., Tillack, H.: Fundus autofluorescence and mfERG for early detection of retinal alterations in patients using chloroquine/hydroxychloroquine. Invest. Ophthalmol. Vis. Sci. **47**, 3531–3538 (2006). 10.1167/iovs.05-1290 16877425 10.1167/iovs.05-1290

[6] Lyons, J.S., Severns, M.L.: Using multifocal ERG ring ratios to detect and follow Plaquenil retinal toxicity: A review. In: Documenta Ophthalmologica. pp. 29–36 (2009) 10.1007/s10633-008-9130-0 18465156

[7] Marmor, M.F., Kellner, U., Lai, T.Y.Y., Melles, R.B., Mieler, W.F., Lum, F.: Recommendations on screening for chloroquine and hydroxychloroquine retinopathy (2016 Revision). Ophthalmology **123**, 1386–1394 (2016). 10.1016/j.ophtha.2016.01.058 26992838 10.1016/j.ophtha.2016.01.058

[8] Dutch society for rheumatology: Viewpoint: (Hydroxy)chloroquine and retinopathy screening. (2018) via: https://www.nvr.nl/wp-content/uploads/2018/12/StandpuntHCQretinopathierevisiehr2018nov.pdf

[9] Dutch Public health data: Reumatoïde artritis (RA) Numbers & Context, via https://www.volksgezondheidenzorg.info/onderwerp/reumato%C3%AFde-artritis-ra/cijfers-context/huidige-situatie#node-prevalentie-reumato%C3%AFde-artritis-huisartsenpraktijk

[10] Rentsch, C.T., DeVito, N.J., MacKenna, B., Morton, C.E., Bhaskaran, K., Brown, J.P., Schultze, A., Hulme, W.J., Croker, R., Walker, A.J., Williamson, E.J., Bates, C., Bacon, S., Mehrkar, A., Curtis, H.J., Evans, D., Wing, K., Inglesby, P., Mathur, R., Drysdale, H., Wong, A.Y.S., McDonald, H.I., Cockburn, J., Forbes, H., Parry, J., Hester, F., Harper, S., Smeeth, L., Douglas, I.J., Dixon, W.G., Evans, S.J.W., Tomlinson, L., Goldacre, B.: Effect of pre-exposure use of hydroxychloroquine on COVID-19 mortality: a population-based cohort study in patients with rheumatoid arthritis or systemic lupus erythematosus using the OpenSAFELY platform. Lancet Rheumatol. **3**, e19–e27 (2021). 10.1016/S2665-9913(20)30378-7 33349815 10.1016/S2665-9913(20)30378-7 PMC7745258

[11] Dutch Ministry of public health and healthcare: Waiting times, via: https://www.vzinfo.nl/wachttijden/ziekenhuiszorg/polikliniek

[12] Dutch Public health data: Rankings - Conditions based on health care expenditures, via: https://www.vzinfo.nl/ranglijsten/aandoeningen-op-basis-van-zorguitgaven

[13] Husereau, D., Drummond, M., Augustovski, F., de Bekker-Grob, E., Briggs, A.H., Carswell, C., Caulley, L., Chaiyakunapruk, N., Greenberg, D., Loder, E., Mauskopf, J., Mullins, C.D., Petrou, S., Pwu, R.F., Staniszewska, S.: Consolidated Health Economic Evaluation Reporting Standards 2022 (CHEERS 2022) statement: updated reporting guidance for health economic evaluations. BMC Med. (2022). 10.1186/s12916-021-02204-0 35014160 10.1111/1471-0528.17012

[14] Dutch Institute National Health Care: Protocol for the execution of economic evaluation in healthcar). 29–02–2016. 120 (2016)

[15] Yusuf, I.H., Sharma, S., Luqmani, R., Downes, S.M.: Hydroxychloroquine retinopathy. Eye (Basingstoke) **31**, 828–845 (2017). 10.1038/eye.2016.298 10.1038/eye.2016.298 PMC5518824 28282061

[16] Melles, R.B., Marmor, M.F.: Rapid macular thinning is an early indicator of hydroxychloroquine retinal toxicity. Ophthalmology **129**, 1004–1013 (2022). 10.1016/j.ophtha.2022.05.002 35568277 10.1016/j.ophtha.2022.05.002

[17] Garrity, S.T., Jung, J.Y., Zambrowski, O., Pichi, F., Su, D., Arya, M., Waheed, N.K., Duker, J.S., Chetrit, Y., Miserocchi, E., Giuffrè, C., Kaden, T.R., Querques, G., Souied, E.H., Freund, K.B., Sarraf, D.: Early hydroxychloroquine retinopathy: Optical coherence tomography abnormalities preceding Humphrey visual field defects. Br. J. Ophthalmol. **103**, 1600–1604 (2019). 10.1136/bjophthalmol-2018-313350 30819690 10.1136/bjophthalmol-2018-313350

[18] Melles, R.B., Marmor, M.F.: The risk of toxic retinopathy in patients on long-term hydroxychloroquine therapy. JAMA Ophthalmol. **132**, 1453–1460 (2014). 10.1001/jamaophthalmol.2014.3459 25275721 10.1001/jamaophthalmol.2014.3459

[19] Melles, R.B., Jorge, A.M., Marmor, M.F., Zhou, B., Conell, C., Niu, J., McCormick, N., Zhang, Y., Choi, H.K.: Hydroxychloroquine dose and risk for incident retinopathy. Ann. Intern. Med. (2023). 10.7326/m22-2453 36645889 10.7326/M22-2453

[20] Statistics, D.: Dutch Statistics, via: https://www.cbs.nl/nl-nl/cijfers/detail/37296ned

[21] Czoski-Murray, C., Carlton, J., Brazier, J., Young, T., Papo, N.L., Kang, H.K.: Valuing condition-specific health states using simulation contact lenses. Value in Health. **12**, 793–799 (2009). 10.1111/j.1524-4733.2009.00527.x 19490557 10.1111/j.1524-4733.2009.00527.x

[22] Mehta, S.: Age-related macular degeneration. Prim. Care **42**, 377–391 (2015). 10.1016/j.pop.2015.05.009 26319344 10.1016/j.pop.2015.05.009

[23] Allahdina, A.M., Chen, K.G., Alvarez, J.A., Wong, W.T., Chew, E.Y., Cukras, C.A.: Longitudinal changes in eyes with hydroxychloroquine retinal toxicity. Retina **39**, 473–484 (2019) 30741731 10.1097/IAE.0000000000002437 PMC12066020

[24] Dutch Statistics: StatLine Annual change in consumer price index; from 1963, via: https://opendata.cbs.nl/statline/#/CBS/nl/dataset/70936ned/table?ts=15210215976

[25] Hakkaart-van Roijen, L., van der Linden, N., Bouwmans, C., Kanters, T., Swan Tan, S.: Kostenhandleiding: Methodologie van kostenonderzoek en referentieprijzen voor economische evaluaties in de gezondheidszorg. Dutch National Health Care Institute. 1–73 (2016)

[26] Elshout, M., van der Reis, M.I., Webers, C.A.B., Schouten, J.S.A.G.: The cost-utility of aflibercept for the treatment of age-related macular degeneration compared to bevacizumab and ranibizumab and the influence of model parameters. Graefe’s Archive for Clinical and Experimental Ophthalmology. **252**, 1911–1920 (2014). 10.1007/s00417-014-2641-3 24777708 10.1007/s00417-014-2641-3

[27] DBC: 079699011.

[28] Brown, M.M., Brown, G.C., Lieske, H.B., Tran, I., Turpcu, A., Colman, S.: Societal costs associated with neovascular age-related macular degeneration in the United States. 10.1097/IAE.0000000000000717 26428606

[29] Dutch statistics (CBS): Labor force participation; key figures seasonally adjusted, via https://www.cbs.nl/nl-nl/cijfers/detail/85224NED?dl=77B4A

[30] Nederlandse Zorg Authoriteit (NZA): DBC-Zorgproducten, https://www.opendisdata.nl/msz/zorgproduct

[31] Briggs, A., Claxton, K., Sculpher, M.: Decision modelling for health economic evaluation. Handbooks in health economic evaluation, Oxford, Oxford (2006)

[32] McClellan, A.J., Chang, J.S., Smiddy, W.E.: Aiming for the bull’s eye: The cost-utility of screening for hydroxychloroquine retinopathy. Retina **36**, 1958–1963 (2016). 10.1097/IAE.0000000000001235 27465574 10.1097/IAE.0000000000001235

