# Supplementary material for: Cost-effectiveness of hydroxychloroquine retinopathy screening: the current guideline versus no screening and reduced regimens
Source: Eur J Health Econ. 2024 Aug 20;26(3):413–25. doi: 10.1007/s10198-024-01715-w (PMC11937206; doi:10.1007/s10198-024-01715-w)
Supplement: Supplementary file 1 — Supplementary file1 (DOCX 19 KB) [file 10198_2024_1715_MOESM1_ESM.docx]

Supporting file 1 – Overview of CHEERS checklist

**Table 1.** Overview of CHEERS checklist and sections where reported

| **Section/topic** | **Item No.** | **Guidance for reporting** | **Reported (in section)** |
| --- | --- | --- | --- |
| **Title** | 1 | Identify the study as an economic evaluation and specify the interventions  being compared | Yes |
| **Abstract** | 2 | Provide a structured summary that highlights context, key methods, results,  and alternative analyses. | Yes |
| **Introduction** | | | |
| **Background and objectives** | 3 | Give the context for the study, the study question, and its practical relevance  for decision making in policy or practice. | Yes |
| **Methods** | | | |
| **Health economic analysis plan** | 4 | Indicate whether a health economic analysis plan was developed and where  available | N/A |
| **Study population** | 5 | Describe characteristics of the study population (such as age range,  demographics, socioeconomic, or clinical characteristics) | Yes (Patient characteristics) |
| **Setting and location** | 6 | Provide relevant contextual information that may influence findings | Yes (Study characteristics) |
| **Comparators** | 7 | Describe the interventions or strategies being compared and why chosen | Yes (Study characteristics and Reduced screening regimens) |
| **Perspective** | 8 | State the perspective(s) adopted by the study and why chosen | Yes (Study characteristics) |
| **Time horizon** | 9 | State the time horizon for the study and why appropriate | Yes (Study characteristics) |
| **Discount rate** | 10 | Report the discount rate(s) and reason chosen. | Yes (Utility values and Costs) |
| **Selection of outcomes** | 11 | Describe what outcomes were used as the measure(s) of benefit(s) and harm(s). | Yes (Study characteristics) |
| **Measurement of outcomes** | 12 | Describe how outcomes used to capture benefit(s) and harm(s) were measured. | Yes (Utilities and Costs) |
| **Valuation of outcomes** | 13 | Describe the population and methods used to measure and value outcomes | Yes (Study characteristics) |
| **Measurement and valuation of resources and costs** | 14 | Describe how costs were valued | Yes (Costs) |
| **Currency, price date, and conversion** | 15 | Report the dates of the estimated resource quantities and unit costs, plus the  currency and year of conversion | Yes (Costs) |
| **Rationale and description of model** | 16 | If modelling is used, describe in detail and why used. Report if the model is  publicly available and where it can be accessed. | Yes (Model structure) |
| **Analytics and assumptions** | 17 | Describe any methods for analysing or statistically transforming data, any  extrapolation methods, and approaches for validating any model used. | Yes (Transition probabilities, utilities, Supporting file 1 and 2) |
| **Characterising heterogeneity** | 18 | Describe any methods used for estimating how the results of the study vary  for subgroups. | Yes (Reduced screening regimens) |
| **Characterising distributional effects** | 19 | Describe how impacts are distributed across different individuals or adjustments  made to reflect priority populations. | Yes (Reduced screening regimens) |
| **Characterising uncertainty** | 20 | Describe methods to characterise any sources of uncertainty in the analysis | Yes (Sensitivity and scenario analyses) |
| **Approach to engagement with patients and others affected by the study** | 21 | Describe any approaches to engage patients or service recipients, the general  public, communities, or stakeholders (such as clinicians or payers) in the design  of the study. | Yes (Patient characteristics) |
| **Results** | | | |
| **Study parameters** | 22 | Report all analytic inputs (such as values, ranges, references) including uncertainty  or distributional assumptions. | Yes (Supporting file 4) |
| **Summary of main results** | 23 | Report the mean values for the main categories of costs and outcomes of interest  and summarise them in the most appropriate overall measure | Yes (Cost-effectiveness of screening guideline in general population) |
| **Effect of uncertainty** | 24 | Describe how uncertainty about analytic judgments, inputs, or projections affect  findings. Report the effect of choice of discount rate and time horizon, if  applicable. | Yes (Probabilistic sensitivity analysis, deterministic sensitivity analysis, scenario analysis) |
| **Effect of engagement with patients and others affected by the study** | 25 | Report on any difference patient/service recipient, general public, community, or  stakeholder involvement made to the approach or findings of the study | N/A |
| **Discussion** | | | |
| **Study findings, limitations, generalisability, and current knowledge** | 26 | Report key findings, limitations, ethical or equity considerations not captured,  and how these could affect patients, policy, or practice | Yes |
| **Other relevant information** | | | |
| **Source of funding** | 27 | Describe how the study was funded and any role of the funder in the  identification, design, conduct, and reporting of the analysis | Yes |
| **Conflicts of interest** | 28 | Report authors conflicts of interest according to journal or International  Committee of Medical Journal Editors requirements | Yes |
