# Supplementary material for: Cost-effectiveness of hydroxychloroquine retinopathy screening: the current guideline versus no screening and reduced regimens
Source: Eur J Health Econ. 2024 Aug 20;26(3):413–25. doi: 10.1007/s10198-024-01715-w (PMC11937206; doi:10.1007/s10198-024-01715-w)
Supplement: Supplementary file 2 — Supplementary file2 (DOCX 23 KB) [file 10198_2024_1715_MOESM2_ESM.docx]

**Supplementary Material 2 – Extrapolated risk for retinopathy measured by Melles et al.**

**Figure 1** outcomes of extrapolation using a polynomial model

**Table 1** outcomes of different risk extrapolation models

| Dose: >5.0 mg/kg/day | | |
| --- | --- | --- |
|  | AIC | BIC |
| Linear | -96.77 | -95.58 |
| Exponential | -92.38 | -91.18 |
| Polynomial | -102.18 | -100.59 |
| Generalized | Inf | Inf |
| Time series | -87.47 | -86.86 |
| Survival  (Most fitting: generalized gamma) | 243.06 | 266.95 |
| Dose: 5.0 – 6.0 mg/kg/day | | |
|  | AIC | BIC |
| Linear | -60.86 | -59.67 |
| Exponential | -63.80 | -62.60 |
| Polynomial | -61.80 | -60.21 |
| Generalized | Inf | Inf |
| Survival  (Most fitting: generalized gamma) | 243.06 | 255.95 |
| Dose: >6.0 mg/kg/day | | |
|  | AIC | BIC |
| Linear | -68.65 | -66.53 |
| Exponential | -95.28 | -93.16 |
| Polynomial | -98.42 | -95.58 |
| Generalized | Inf | Inf |
| Survival  (Most fitting: generalized gamma) | 243.06 | 255.951 |
| **Note**: a lower AIC and BIC indicates a better fitting the model **Abbreviations:** AIC; Akaike information criterion BIC; Bayesian information criterion | | |
