# Supplementary material for: Cost-effectiveness of hydroxychloroquine retinopathy screening: the current guideline versus no screening and reduced regimens
Source: Eur J Health Econ. 2024 Aug 20;26(3):413–25. doi: 10.1007/s10198-024-01715-w (PMC11937206; doi:10.1007/s10198-024-01715-w)
Supplement: Supplementary file 3 — Supplementary file3 (DOCX 13 KB) [file 10198_2024_1715_MOESM3_ESM.docx]

**Supplementary material 3 – Least ordinary square model used to calculate utility scores**

**Table 1** input parameters to determine quality of life based on visual acuity and age

|  | Input |
| --- | --- |
| Constant | 0.860 (0.068) |
| Visual acuity LogMAR | -0.368 (0.046) |
| Age | -0.001 (0.002) |
| Adjusted R^2^ | 0.172 |
