# Supplementary material for: Cost-effectiveness of hydroxychloroquine retinopathy screening: the current guideline versus no screening and reduced regimens
Source: Eur J Health Econ. 2024 Aug 20;26(3):413–25. doi: 10.1007/s10198-024-01715-w (PMC11937206; doi:10.1007/s10198-024-01715-w)
Supplement: Supplementary file 4 — Supplementary file4 (DOCX 13 KB) [file 10198_2024_1715_MOESM4_ESM.docx]

**Appendix 4 – model assumptions**

**Table A1** model assumptions

| **Model assumptions** |
| --- |
| Patients develop severe retinopathy after 3 years of undetected retinopathy. |
| Patients with severe retinopathy are assumed to have vision loss corresponding with the most severe state of retinopathy and their vision loss remains stable. |
| The risk of retinopathy is based on the yearly incidence based on dosage found in the study of Melles et al ^18^ |
| QALYs are based on sight and age ^20^. |
| Patients are screened yearly after developing stabilized and severe retinopathy. |
| **Abbreviations:** QALYs: Quality-adjusted life-years |
