# Supplementary material for: Cost-effectiveness of hydroxychloroquine retinopathy screening: the current guideline versus no screening and reduced regimens
Source: Eur J Health Econ. 2024 Aug 20;26(3):413–25. doi: 10.1007/s10198-024-01715-w (PMC11937206; doi:10.1007/s10198-024-01715-w)
Supplement: Supplementary file 5 — Supplementary file5 (DOCX 15 KB) [file 10198_2024_1715_MOESM5_ESM.docx]

**Supplementary material 5 – model parameters and their distributions**

Table 1 model parameters, distribution, and distribution type

| **Parameter** | **Distribution** | **Distribution type** |
| --- | --- | --- |
| Average age | 46.20 (27.60-64.80) | Normal |
| Risk for retinopathy | - | Beta |
| Sensitivity SD-OCT + HFA | 0.86 (0.23-1.00) | Beta |
| Sensitivity SD-OCT | 0.79 (0.30-0.99) | Beta |
| Costs screening SD-OCT + HFA^a^ | 175 (100-271) | Gamma |
| Costs screening HFA^a^ | 38 (59-21) | Gamma |
| Average costs vision aids for visual acuity 0.7 LogMAR^a^ | 1020 (583-1,577) | Gamma |
| Travel costs per km | 0.19 (0.11-0.29) | Gamma |
| Average hospital distance | 7 (4-10.8) | Gamma |
| Workforce Netherlands (male) | 0.67 (0.31-0.94) | Beta |
| Workforce Netherlands female | 0.56 (0.28-0.81) | Beta |
| Workforce retinopathy | 0.06 (0.03-0.08) | Beta |
| Average work week (hours) | 32.1 (18.3-49.6) | Gamma |
| Friction period (days) | 85 (49-131) | Gamma |
| Hourly wage^a^ | 34.8 (19.9-131.4) | Gamma |
| Opportunity costs informal care^a^ | 14.0 (8.0-21.7) | Gamma |
| Yearly hours of informal care after retinopathy | 127 (73-196) | Gamma |
| Average screening duration (OCT + HFA) | 1 (0.6-1.6) | Gamma |
| Average screening duration (OCT) | 0.5 (0.3-0.8) | Gamma |
| LogMAR visual acuity without retinopathy | 0.07 (0.00-0.18) | Beta |
| LogMAR visual acuity with severe retinopathy | 0.7 (0.50-0.90) | Beta |
| QoL correction for vision loss | 0.37 (0.20-0.56) | Beta |
| QoL correction for age | 0.001 (0.00-0.002) | Beta |
| Key: ^a^ All costs reported in this table are the cost inputs before correction for inflation.  Abbreviations: HCQ: Hydroxychloroquine, HFA: Humphrey field analyzer, LogMAR: logarithmic minimum angle of resolution, SD-OCT: spectral-domain optical coherence tomography QALYs: quality-adjusted life-years, QoL: Quality of life | | |
