# Supplementary material for: Cost-effectiveness of hydroxychloroquine retinopathy screening: the current guideline versus no screening and reduced regimens
Source: Eur J Health Econ. 2024 Aug 20;26(3):413–25. doi: 10.1007/s10198-024-01715-w (PMC11937206; doi:10.1007/s10198-024-01715-w)
Supplement: Supplementary file 6 — Supplementary file6 (DOCX 25 KB) [file 10198_2024_1715_MOESM6_ESM.docx]

**Supplementary material 6 – outcomes improved screening regimen per risk group**

**Table 1.** Incremental costs, incremental QALYs, and ICER for several optimized screening regimens compared in a step-wise approach in patients that receive <5.0 mg/kg HCQ per day, 5.0-6.0 mg/kg HCQ per day, and >6.0 mg/kg HCQ per day.

| **Patients that receive <5.0 mg/kg HCQ per day** | | | | | | |
| --- | --- | --- | --- | --- | --- | --- |
| **Proposed option** | **Total costs** | **Total QALYs** | | **Compared to** | **ICER** | **Decision** |
| Current screening guideline | €3,710 | 22.46 | | 10 years increment, SD-OCT only, biannual | €16,709 | Comparator more cost-effective |
| 10 years increment, SD-OCT only, biannual | €1,751 | 22.34 | | 20 years increment, SD-OCT only, biannual | Dominant | Comparator disregarded |
|  |  |  |  | 15 years increment, SD-OCT only, biannual | Dominant | Comparator disregarded |
|  |  |  |  | 20 years increment, SD-OCT only | Dominant | Comparator disregarded |
|  |  |  |  | 15 years increment, SD-OCT | €38,313 | Proposed option most cost-effective |
| 20 years increment, SD-OCT only, biannual | €1,814 | 22.25 | | N/A | | |
| 15 years increment, SD-OCT only, biannual | €2,014 | 22.30 | | N/A | | |
|  |  |  |  | N/A | | |
| 20 years increment, SD-OCT only | €2,087 | 22.28 | | N/A | | |
| 15 years increment, SD-OCT only | €2,122 | 22.35 | | N/A | | |
|  |  |  |  | N/A | | |
| 20 years increment | €2,379 | 22.29 | | N/A | | |
| 15 years increment | €2,563 | 22.36 | | N/A | | |
| SD-OCT only | €2,824 | 22.43 | | N/A | | |
| 10 years increment | €2,926 | 22.42 | | N/A | | |
| **Patients that receive 5.0-6.0 mg/kg HCQ per day** | | | | | | |
| **Proposed option** | **Total costs** | **Total QALYs** | | **Compared to** | **ICER** | **Decision** |
| Current screening guideline | €3,222 | 22.43 | | 10 years increment, SD-OCT only, biannual | €2,711 | Comparator more cost-effective |
| 10 years increment, SD-OCT only, biannual | €2,599 | 22.20 | | 10 years increment, SD-OCT only | €161 | Comparator more cost-effective |
| 10 years increment, SD-OCT only | €2,623 | 22.35 | | 5 years increment, SD-OCT only, biannual | Dominant | Comparator disregarded |
|  |  |  |  | 15 years increment | Dominant | Comparator disregarded |
|  |  |  |  | 5 years increment, SD-OCT only | €6,510 | Comparator more cost-effective |
| 5 years increment, SD-OCT only, biannual | €2,691 | 22.24 | | N/A |  |  |
| 15 years increment | €2,948 | 22.21 | | N/A |  |  |
| 5 years increment, SD-OCT only | €2,953 | 22.40 | | 15 years increment, SD-OCT only | Dominant | Comparator disregarded |
|  |  |  |  | 10 years increment | Dominant | Comparator disregarded |
|  |  |  |  | 20 years increment | Dominant | Comparator disregarded |
|  |  |  |  | SD-OCT only | Dominant | Comparator disregarded |
|  |  |  |  | 5 years increment | €25,336 | Proposed option most-cost-effective |
| 15 years increment, SD-OCT | €3,047 | 22.19 | | N/A | | |
| 10 years increment | €3,196 | 22.37 | | N/A | | |
| SD-OCT only | €3,547 | 22.40 | | N/A | | |
| 20 years increment | €3,667 | 21.10 | | N/A | | |
| 5 years increment | €3,777 | 22.43 | | N/A | | |
| **Patients that receive >6.0 mg/kg HCQ per day** | | | | | | |
| **Proposed option** | **Total costs** | **Total QALYs** | | **Compared to** | **ICER** | **Decision** |
| Current screening guideline | €4,686 | 22.41 | | 5 years increment, SD-OCT only, biannual | €8,789 | Comparator more cost-effective |
| 5 years increment, SD-OCT only, biannual | €2,806 | 22.20 | | 10 years increment, SD-OCT only | €855 | Comparator more cost-effective |
| 10 years increment, SD-OCT only | €2,891 | 22.30 | | 5 years increment, SD-OCT only | €303 | Comparator more cost-effective |
| 5 years increment, SD-OCT only | €3,194 | 22.36 | | 10 years increment | Dominant | Comparator disregarded |
|  |  |  |  | 10 years increment, SD-OCT only, biannual | Dominant | Comparator disregarded |
|  |  |  |  | SD-OCT only | €49,516 | Proposed option most cost-effective |
| 10 years increment | €3,381 | 22.34 | | N/A | | |
| 10 years increment, SD-OCT only, biannual | €3,613 | 22.03 | | N/A | | |
| SD-OCT only | €3,703 | 22.37 | | N/A | | |
| 5 years increment | €3,896 | 22.36 | | N/A | | |
| 15 years increment, SD-OCT only | €4,325 | 21.96 | N/A | | | |
| 15 years increment | €4,638 | 21.99 | N/A | | | |
| 20 years increment | €6,796 | 21.49 | N/A | | | |
| **Abbreviations:** HCQ: Hydroxychloroquine, HFA: Humphrey Field Analyzer, ICER: Incremental cost-effectiveness ratio SD-OCT: Spectral domain optical coherence tomography, QALYs: Quality-adjusted life-year | | | | | | |
