# Supplementary material for: Cost-effectiveness of hydroxychloroquine retinopathy screening: the current guideline versus no screening and reduced regimens
Source: Eur J Health Econ. 2024 Aug 20;26(3):413–25. doi: 10.1007/s10198-024-01715-w (PMC11937206; doi:10.1007/s10198-024-01715-w)
Supplement: Supplementary file 7 — Supplementary file7 (DOCX 22 KB) [file 10198_2024_1715_MOESM7_ESM.docx]

**Supporting file 7 – tornado diagrams presenting the outcomes of the deterministic sensitivity analysis**

**Figure 1** Tornado diagrams presenting the most influential model parameters for the cost-effectiveness of screening compared to no screening in the general population.
**Key**: The upper bound represents the outcomes for the maximum value that used in the deterministic sensitivity analysis. The lower bound represents the outcomes for the minimum value that is used in the deterministic sensitivity analysis.
**Abbreviations:** HCQ: Hydroxychloroquine, HFA: Humphrey Field Analyzer, SD-OCT: Spectral domain optical coherence tomography, LogMAR: Logarithm of the Minimum Angle of Resolution, QALYs: quality-adjusted life years
